# The Smoky Stick Insect: Defensive Chemicals From a Vietnamese Phasmatodean (Necrosciinae: *Neohirasea catbaensis*)

**DOI:** 10.1007/s10886-026-01754-y

**Published:** 2026-09-01

**Authors:** Marco Niekampf, Till Beuerle, Paul Meyer, Sven Bradler, Thomas Schmitt

**Affiliations:** 1 Biological Collection, Bielefeld University, Universitätsstraße 25, Bielefeld, 33615 Germany; 2 Department of Animal Evolution and Biodiversity, Johann-Friedrich-Blumenbach Institute of Zoology and Anthropology, University of Göttingen, Untere Karspüle 2, Göttingen, 37073 Germany; 3 Institut für Pharmazeutische Biologie, Technische Universität Braunschweig, Mendelssohnstrasse 1, Braunschweig, 38106 Germany; 4 Institute for X-Ray Physics, University of Göttingen, Friedrich-Hund-Platz 1, Göttingen, 37077 Germany; 5 Department of Animal Ecology and Tropical Biology, Biocenter, University of Würzburg, Am Hubland, Würzburg, 97074 Germany

**Keywords:** Insect chemical defence, Defensive strategy, Secondary defence, 4-vinylphenol

## Abstract

Beyond their impressive repertoire of camouflage as the predominant primary defensive strategy, stick and leaf insects (Phasmatodea) can actively defend themselves chemically with deterrent substances emitted from a pair of prothoracic repellent glands. The Vietnamese Prickly Stick Insect *Neohirasea catbaensis* emits a conspicuous secretion when disturbed, often described as smoky, which constitutes a unique characteristic not known from any other stick and leaf insect. Here we investigate the anatomy and the chemical components of the repellent glands in this species. Via micro-computed tomography (µ-CT), we observed comparatively small sac-like glands with no ejaculatory ducts present. The anatomy of the repellent glands of both male and female is described in detail. By applying gas chromatography (GC) in combination with high-resolution accurate mass spectrometry (HRAM-MS), we found 4-vinylphenol as the major component of the secretion, accompanied by 2-methoxy-4-vinylphenol and eugenol as minor constituents. This is the first time that these molecules have been identified as repellent substances from an animal. We discuss these findings in relation to the chemical diversity and ecological function of phasmatodean repellent secretions.

## Introduction

**Fig. 1:**
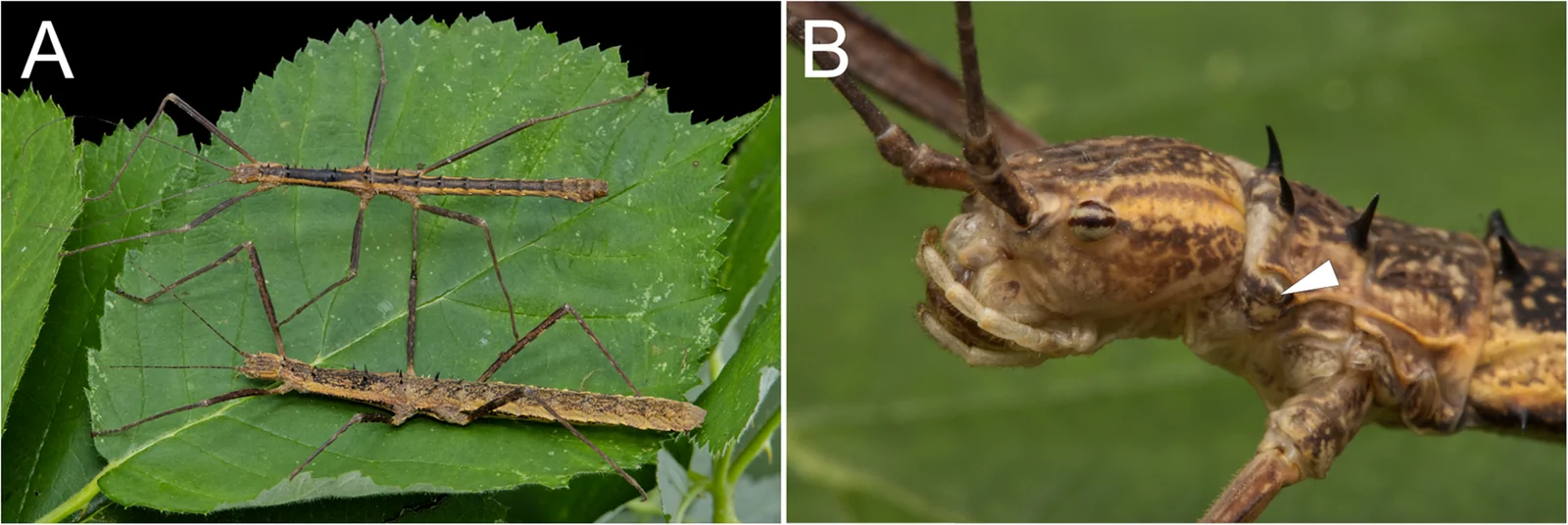
Photographs of *Neohirasea catbaensis*. **A**: couple, male above female. **B**: female head and prothorax with the repellent gland opening (arrowhead)

Chemical defense against predators and parasitoids is used by a variety of insect taxa such as earwigs (Dermaptera), grasshoppers and bushcrickets (Orthoptera), cockroaches (Blattodea), true bugs (Hemiptera), bees, wasps and ants (Hymenoptera), lacewings (Neuroptera), beetles (Coleoptera) and also stick and leaf insects (Phasmatodea, Fig. 1), and repellent secretions are applied through a wide range of morphological features (Eisner et al. 2005). Stick and leaf insects are well-known for a highly effective primary defensive strategy: camouflage via impressive imitation of plant parts such as twigs, leaves, bark and moss (Bedford 1978), yet nearly all known taxa of the 3400 + described species possess a pair of prothoracic glands in different shapes and sizes as part of their secondary defensive repertoire (Niekampf et al. 2024). The chemical composition of only a dozen phasmatodean taxa, mostly unrelated to each other, is known (Dossey 2011; Dossey et al. 2012). These substances, experienced by humans as irritating and malodorous, are released through a pair of openings at the dorsolateral edges of the prothorax and are effective repellents against a variety of predators and parasitoids (Eisner 1965; Strong 1975). In addition, several defensive substances of stick and leaf insects possess antimicrobial and antibacterial properties (Dossey 2010). It is unknown whether there are further effects or functions associated with these glands’ secretions.

Reported secretions may contain substances as diverse as alkylpyrazines, monoterpene alkaloids, monoterpene cyclopentanoids, heteroaromatic compounds, or spiroketals (Dossey 2011; Dossey et al. 2012). Comprehensive lineage-specific analyses are currently lacking. The chemical constituents have mainly been described for individual species: Quinoline from the Peruvian Firestick *Oreophoetes peruana* (Eisner et al. 1997), 4-methyl-1-hepten-3-one from the Chilean phasmid *Agathemera elegans* (Schmeda-Hirschmann 2006), the monoterpenes anisomorphal and dolichodial from the Florida Devil Rider *Anisomorpha buprestoides* (Meinwald et al. 1962; Eisner 1965; Dossey et al. 2006), its stereoisomer peruphasmal from the closely related *Peruphasma schultei* (Dossey et al. 2006), alkyldimethyl pyrazines from the leaf insect *Cryptophyllium westwoodii* (Dossey et al. 2009), parectadial from the Malagasy *Parectatosoma mocquerysi* (Dossey et al. 2007), iridodial and nepetalactone from the Coconut Stick Insect *Graeffea crouani* (Smith et al. 1979), actinidine from Peppermint Stick Insects of the Australopacific genus *Megacrania* (Chow and Lin 1986; Ho and Chow 1993; Prescott et al. 2009; Kobayashi et al. 2023), and in Southeast Asian Necrosciinae stick insects: acetic acid, benzaldehyde, benzothiazole, diethyl ether and limonene from *Sipyloidea chlorotica* (formerly known as *S. sipylus*) (Bouchard et al. 1997) or spiroketals known from *Asceles glaber* (Dossey et al. 2012). Here we describe the repellent gland anatomy and further defensive substances from another member of the Necrosciinae, the Vietnamese Prickly Stick Insect *Neohirasea catbaensis* (Fig. 1, bred in captivity for several decades under the name *Neohirasea maerens*), using micro-computed tomography (µ-CT) and gas chromatography (GC) coupled with high-resolution accurate-mass mass spectrometry (HRAM-MS).

## Methods and Materials

### Specimens

Male and female individuals from lab cultures of the Department of Animal Evolution and Biodiversity, University of Göttingen, were used in this study. The animals were kept in glass terrariums (30 cm x 30 cm x 40 cm) at room temperature, with approximately five females and five males per enclosure. They were sprayed with water two to three times a week to ensure increased humidity and fed with the leaves of *Rubus* spp. (bramble, Rosaceae). Single individuals were alternatively fed with *Quercus* spp. (oak, Fagaceae), *Hypericum* spp. (St. John’s wort, Hypericaceae) and *Eucalyptus* spp. (Myrtaceae) to test whether defensive substances vary with diet.

### µ-CT Specimen Preparation

To avoid the emission of repellent secretion in the dissection process, the animals were carefully cooled for 30 min in small plastic containers with lids in the refrigerator (4 °C). Afterwards, a laboratory paper towel moistened with approximately five droplets of ethyl acetate was added to anaesthetize the insect. The legs and antennae were removed with scissors, and the animals were cut posterior the prothorax, leaving head and thorax for the tomography scan. After 70 h of fixation in 15 ml polypropylene tubes containing 70% Bouin’s solution, the animals were stored for 18 h in the same containers in 1% iodine solution for contrast enhancement. The object was dried via a Balzer CPD030 critical-point dryer.

### Imaging and Image Data Processing

The specimens were glued vertically on specimen stubs (Agar Scientific 0.5”) and scanned in the EasyTom µ-CT system (RX Solutions, France) featuring a sealed X-ray tube (Hamamatsu L12161-07) equipped with a tungsten (W) target and offering a spot size of 5 μm in small focal spot mode. Projection images were captured using a CCD detector (Gadox-scintillator). For precise experimental details of each scan see Table 1.

**Table 1:** µ-CT scan parameters and experimental details of both male and female *Neohirasea catbaensis*

	Tube voltage (kV)	Geometric magnification	Voxel size (µm)	Number of acquisition angles	Accumulation time (s)	Total scan time (h) (+−10 min)	Acquisition time per angle	Detector pixelsize (µm)	Source-object-distance (mm)	Source-detector-distance (mm)
*N. catbaensis male*	80	5	3.53	1568	6	3.2	3 × 2s	18	26.3	134.2
*N. catbaensis* female	60	8	2.30	1568	6	3.0	3 × 2s	18	14.6	114.7

Image processing on the reconstructed volumes was performed with Amira 2021.1. Glands and the digestive system were labeled and afterwards processed with the Biomedisa semi-automatic segmentation platform (Lösel et al. 2020). 3D visualizations were subsequently processed with Affinity Photo 2.6.5 and Affinity Designer 2.6.5.

Living animals were photographed with a Canon EOS90D camera and a Canon EF 100 mm f/2.8 L Macro IS USM Lens.

### Gland Volume and Prothorax Volume Measuring

Due to body size differences between male and female, the absolute glandular size might differ distinctly between sexes. To obtain the relative gland size, the glandular volume was set in relation to the prothorax volume to provide a reference value (gland-prothorax ratio, gpr) for gland size comparisons. This method is outlined in detail in Niekampf et al. (2024).

### Secretion Sampling

To stimulate the emission of the defensive secretion, the animals were grasped by the abdomen and legs while a glass vial (CZ Trott, 1.5 ml) was held over the glandular opening. Following the emission of the secretion, dichloromethane (Roth, Rotisolv GC ultra grade) was added to the vials, filling the vial to approximately 300 µl. A total of 26 samples were collected: ten from male and female individuals each that were fed exclusively on bramble leaves, and three samples from each sex that were fed with a combination of *Quercus*, *Hypericum* and *Eucalyptus*.

### Gas Chromatography-Mass Spectrometry (GC-MS)

The defensive secretion extracts were analyzed with an Agilent 7890B series gas chromatograph coupled to an Agilent 5977 mass selective detector. The analytical process incorporated an HP-5MS column, with dimensions measuring 30 m in length, an inner diameter of 0.25 mm, and a film thickness of 0.25 μm, all from Agilent Technologies, Santa Clara, CA, USA. Automatic injection of 1 µl per extract was executed via the split/splitless injector, operating in splitless mode for 1 min at a constant temperature of 300 °C. Initiated at 60 °C, a gradual temperature increase was applied to the gas chromatograph, with a progression rate of 5 °C per minute. This ascent continued until a temperature of 300 °C was achieved, which was maintained for a duration of 10 min. Helium as carrier gas with a column flow of 1 ml per minute persisted throughout the entire analytical procedure. Parameters for the mass spectrometer encompassed an electron beam energy of 70 eV, a source temperature of 230 °C, and a quadrupole temperature of 150 °C.

Data acquisition and peak area integration were facilitated by employing the MSD ChemStation Data Analysis Application program (F.01.03.2357, Agilent Technologies, Santa Clara, CA, USA). Compounds were examined by comparing their mass spectra and the calculated retention indices with data from a commercially available spectra library (NIST14). For the definite identification of compounds, we compared our data with authentic reference compounds 4-vinylphenol (10 wt% in propylene glycol) and 2-methoxy-4-vinylphenol (99%) as well as eugenol (99%), obtained from BLD Pharmatech GmbH (Reinbek, Germany) and Merck (Darmstadt, Germany). In order to prepare 4-vinylphenol for GC-MS analysis, it was extracted from a solution of the product in water, using dichloromethane.

### Gas Chromatography-High Resolution Accurate Mass Spectrometry (GC-HRAM-MS)

A Thermo Scientific Trace 1310 gas chromatograph (Thermo Scientific, Bremen, Germany) was equipped with a 30 m analytical column (Phenomenex ZB5-MS, 30 m x 0.25 mm ID, t_f_=0.25 μm). A split/splitless injection port at 270 °C was used for sample introduction in split mode (ratio: 1:5). The temperature program was: 50 °C (3 min)−10 °C/min-310 °C (3 min). The helium carrier gas was set to 1.0 ml/min flow rate (constant flow mode).

An Exactive GC orbitrap mass spectrometer (Thermo Scientific, Bremen, Germany) was used as detector. The resolution was set to 60.000 (FWHM; instrument setting at 200 u). The mass range was 45–450 u and 2 micro scans were averaged per data scan. The automated gain control (AGC target) was set to 1 × 10^6^ and maximum inject time was set to “auto”. The Auxiliary temperatures were set to 270 °C for both transfer lines 1 and 2 and the electron ionization source temperature was set to 220 °C. EI was performed at 70 eV energy in positive mode. Helium (carrier gas) and nitrogen (supply for the C-Trap) were equipped with gas purification cartridges to trap moisture and organic impurities of the gases (Thermo Scientific, Bremen, Germany). The column bleed ion at 207.03235 u was used as the lock mass for internal mass calibration of the data. The linear retention indices were calculated with a reference set of hydrocarbons (Sigma-Aldrich).

## Results

### Gland Anatomy

**Fig. 2:**
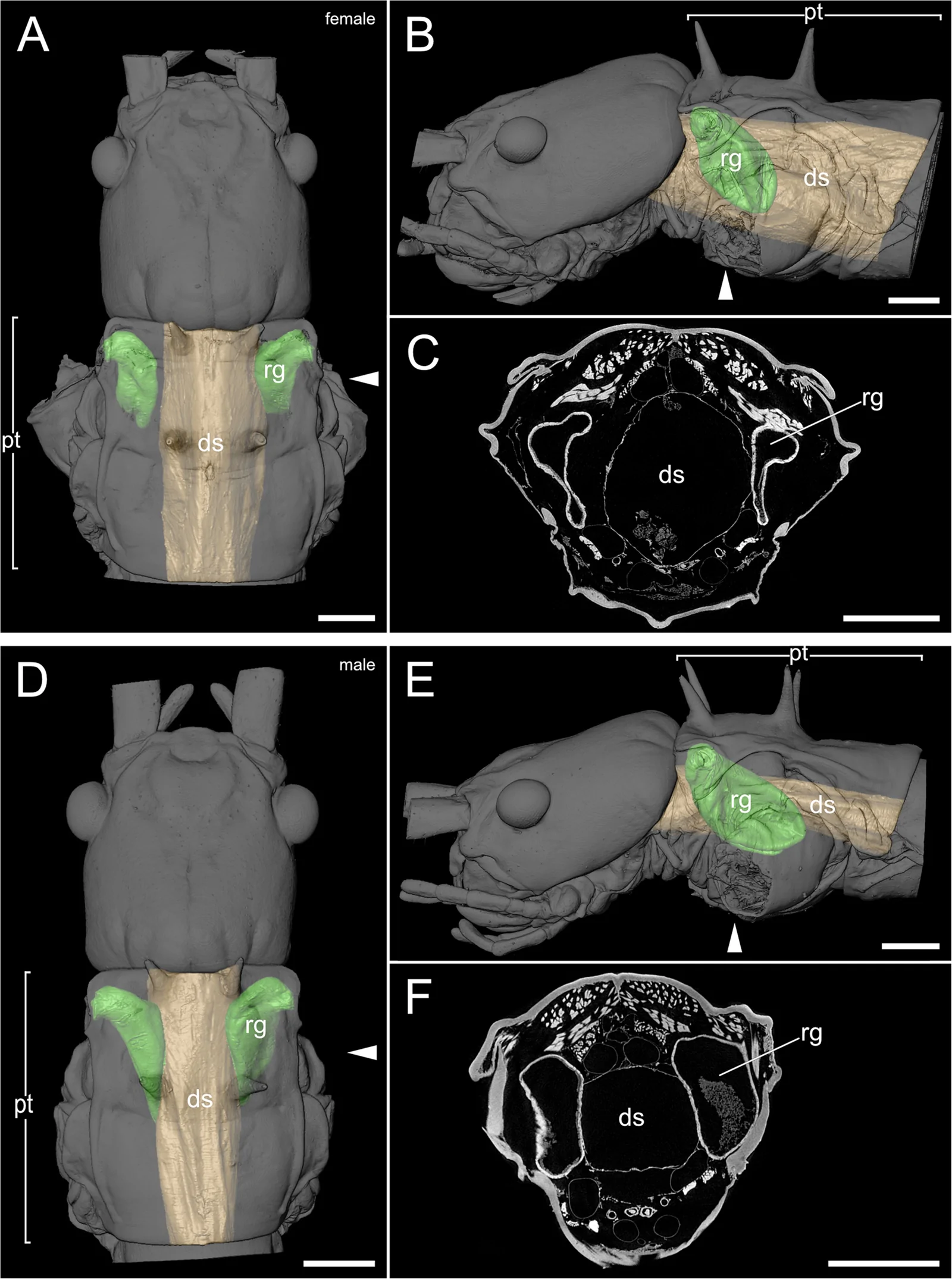
3D visualization and μCT scan cross section of *Neohirasea* *catbaensis* female (**A**–**C**) and male (**D**–**F**). **A**&**D**: dorsal view. B&E: lateral view. **C**&**F**: μCT scan. ds = digestive system, pt = prothorax, rg = repellent gland, arrowhead = area of μCT scan. Scale bars: 1 mm

The µCT scans revealed uniformly structured repellent glands in male and female *Neohirasea catbaensis* (Fig. 2). Previous comparative analyses identified four distinct gland types (Niekampf et al. 2024): (1) lobe-like glands, (2) sac-like glands without ejaculatory duct, (3) sac-like glands with ejaculatory duct, and (4) tube-like glands. We identified type 2 sac-like glands without ejaculatory duct for *N. catbaensis*, consisting of small pouches, no longer than 2 mm that are attached directly to the glandular opening. The average absolute volumes of both glands in male and female are approximately the same, however the gland-prothorax ratios (gpr) differ. The female and male have an average gland size of 0.77 mm³ (female both glands 0.77 mm³, male left gland 0.75 mm³ and right gland 0.79 mm³) while the gpr is 3.7% in females and 8.7% in males, thus males have relatively larger glands compared to their body size.

### Gland Chemistry

**Fig. 3:**
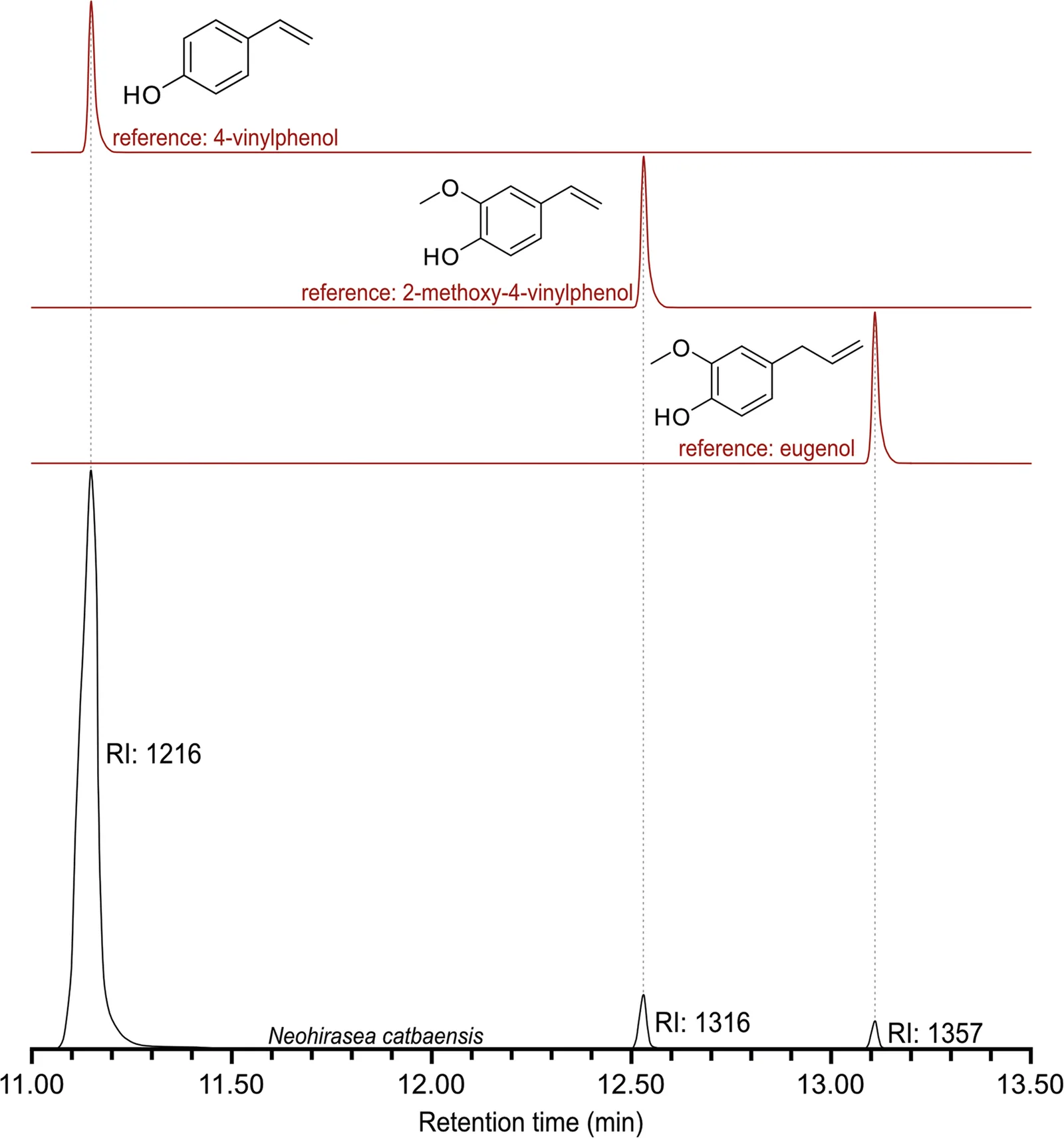
Representative GC-MS chromatogram of a secretion sample from* Neohirasea catbaensis* and the authentic reference compounds of 4-vinylphenol, 2-methoxy-4-vinylphenol and eugenol. RI = retention index

**Fig. 4:**
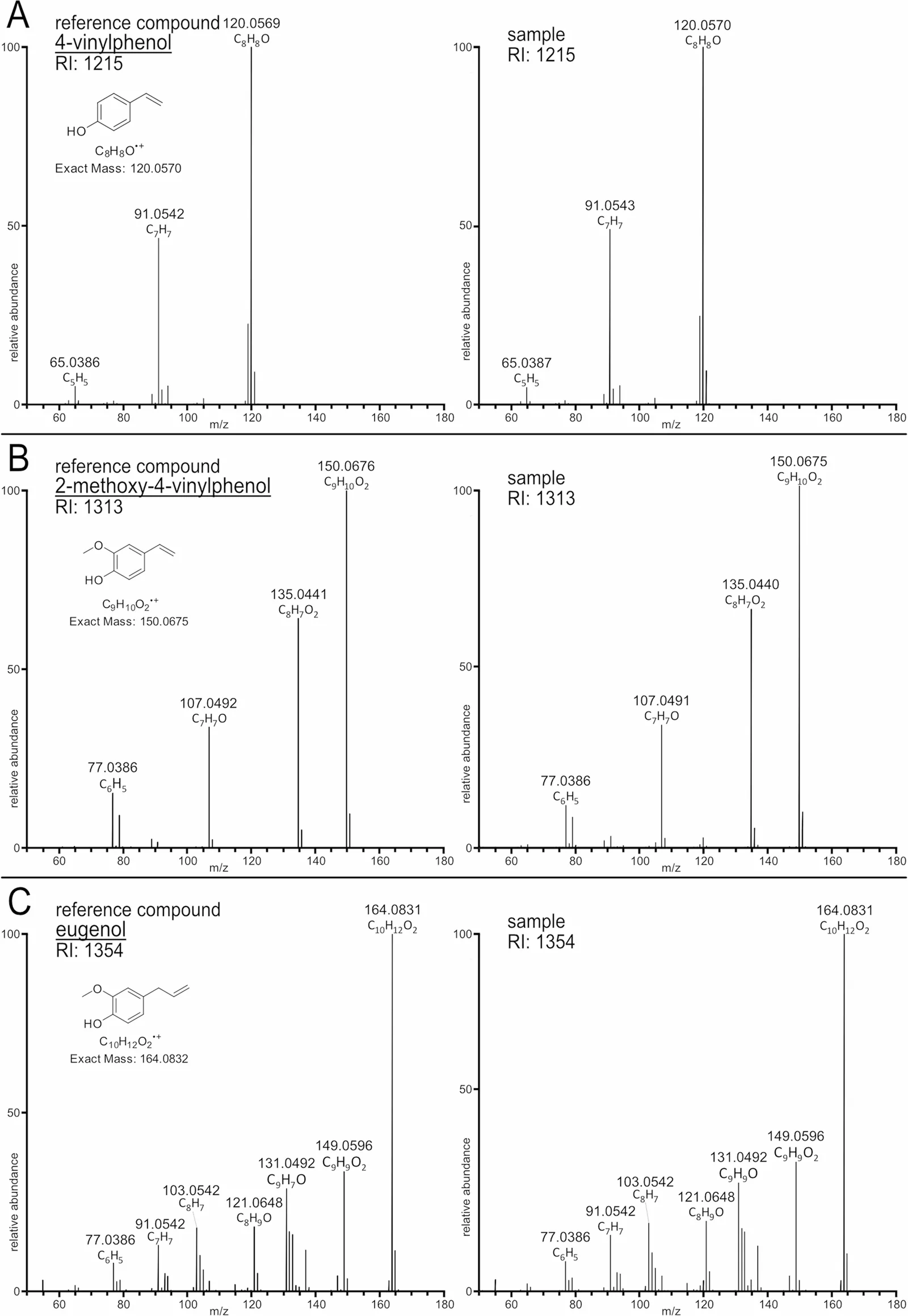
HRAM mass spectra of the reference compounds (left) and secretion samples of *Neohirasea catbaensis* (right) with the respective components. A: 4-vinylphenol. B: 2-methoxy-4-vinylphenol. C: eugenol. RI = retention index

We identified three substances in the repellent secretion of *N. catbaensis*: 4-vinylphenol, 2-methoxy-4-vinylphenol and eugenol (2-methoxy-4-(prop-2-en-1-yl)-phenol). 4-vinylphenol was always present as major component with a retention index of 1216 (Fig. 3). 2-methoxy-4-vinylphenol and eugenol were identified as minor components with retention indices of 1316 and 1357. The utilization of a slightly different type of a DB-5-type column in the GC-HRAM-MS analysis (ZB5-MS instead of HP5-MS) explains the observation of slightly different retention indices, with 1215 in 4-vinylphenol, 1313 in 2-methoxy-4-vinylphenol and 1354 in eugenol (Fig. 4). In females, 4-vinylphenol accounted for an average of 94% in the defensive secretion, 2-methoxy-4-vinylphenol for 5%, and eugenol for 1% (Table 2). In males, the average was 95% 4-vinylphenol, 4% 2-methoxy-4-vinylphenol, and 1% eugenol. All substances exhibit matching retention indices, chromatograms and mass spectra compared to the authentic reference compounds (Figs. 3 and 4). 4-vinylphenol was present in all samples, whereas the other two substances were not always detected, but 2-methoxy-4-vinylphenol was always found in higher quantities than eugenol. In seven samples from females and nine samples from males, 2-methoxy-4-vinylphenol and eugenol were not detected and exclusively 4-vinylphenol was identified. The chemical analyses of individuals fed varying types of foodplants consistently revealed the same three compounds in similar relative proportions, regardless of diet.

**Table 2:** Average proportions with standard deviation (± SD) in male and female *Neohirasea catbaensis*, based on peak integrations

	Female			Male		
	4-vp	2-m-4-vp	eug	4-vp	2-m-4-vp	eug
Average	94% (± SD 0.06)	5% (± SD 0.06)	1% (± SD 0.06)	95% (± SD 0.06)	4% (± SD 0.06)	1% (± SD 0.06)

## Discussion

Defensive strategies in stick and leaf insects are remarkably diverse but still poorly understood. By integrating gland morphology and chemical analyses, this study uncovers the defensive compounds and morphological features in *Neohirasea catbaensis.* The sac-like glands without ejaculatory duct, present in both male and female (Fig. 2) are relatively small compared to the only other Necrosciinae taxon anatomically studied so far: The Yellow Flying Stick *Anarchodes annulipes*, which possess large tube-like glands measuring approximately 13 mm in length, some of the largest known for Phasmatodea (Niekampf et al. 2024). Other Necrosciinae might exhibit smaller glands. In the Malagasy *Sipyloidea chlorotica*, gland length was reported as approximately 1.5 mm by Bouchard et al. (1997), however, without any detailed morphological data like CT-based analyses or comparable imaging. The significance of gland size is not well understood so far, and the conclusion that larger glands indicate a more effective chemical defense appears to be premature: Two well-studied species exhibiting prominent spraying capabilities associated by conspicuous aposematic coloration, the Florida Devil Rider *Anisomorpha buprestoides* and the Peruvian Fire Stick *Oreophoetes peruana* possess glands of significantly different sizes, with *Oreophoetes* having small glands producing the aromatic quinoline (Eisner et al. 1997; Niekampf et al. 2024) and *Anisomorpha* having very large glands producing the monoterpene anisomorphal (Meinwald et al. 1962; Dossey et al. 2006). While anisomorphal has been tested to successfully repel various predators ranging from ants to birds (Eisner 1965), nothing is known about the impact of most other defensive compounds so far, including those of *N. catbaensis*. Given the limited data set, the evolutionary aspects underlying gland sizes and defensive substances remain unclear and numerous ecological and physiological factors might influence these traits in Phasmatodea. In the male, the repellent glands are larger relative to the prothorax, with the male gland-prothorax ratio being more than twice the size compared to the female. The relative differences between the sexes cannot be explained by different chemical compounds, since males produce the same substances as females. *N. catbaensis* exhibits a pronounced sexual dimorphism, with males being considerably smaller (Fig. 1) and more mobile than females, a common pattern in stick and leaf insects (Bradler and Buckley 2018; Boisseau et al. 2020). Given their smaller body size, their proportionally enlarged glands appear to compensate for the size differences, resulting in an absolute gland volume comparable to that of females, since both sexes are likely exposed to the same predator community.

The three repellent substances identified in *N. catbaensis* have, to our knowledge, not previously been reported from any other animal. Stick and leaf insects exhibit a remarkable chemical diversity in their defensive secretions, with nearly every studied species producing a distinct set of compounds (summarized in the introduction). *N. catbaensis* and the only two other Necrosciinae species investigated so far *Sipyloidea chlorotica*, and *Asceles glaber*, rely on different substances for defense. *Asceles* produces spiroketals (Dossey et al. 2012), *Sipyloidea* uses a mixture of diethyl ether, benzothiazole, and benzaldehyde (Bouchard et al. 1997), and *Neohirasea* employs yet another unique combination of chemicals, demonstrating that even closely related species evolved highly divergent chemical repertoires. The Necrosciinae are considered the most species-rich and diverse lineage considered as subfamily (Bradler et al. 2014) within the stick and leaf insects, and the current limited taxon sampling already indicates a striking variety in defensive chemistry that needs to be further explored.

All three substances identified in *N. catbaensis* are already well known from plants, fungi, and bacteria (Heravi et al. 2017; Marchese et al. 2017). 4-vinylphenol is present in plants like wild rice (*Zizania aquatica*) (Withycombe et al. 1978) and Chinese medicinal herb (*Hedyotis diffusa*) (Chen et al. 2016), and is formed during the fermentation in wine and beer by yeasts such as *Saccharomyces cerevisiae* and *Dekkera bruxellensis* (Chatonnet et al. 1993; Godoy et al. 2009). Moreover, 4-vinylphenol can cause pneumotoxicity and hepatotoxicity in mice and, at certain concentrations, even lead to the death of the animals. After an intraperitoneal injection of 200 mg 4-vinylphenol per kilogram body weight, the mice died within 40 h (Carlson et al. 2002). This makes 4-vinylphenol the first known defensive substance of stick and leaf insects that not (only) has an irritating and/or malodorous effect but also shows toxic activities. However, the quantities required to kill a potential predator most probably exceed those released by *N. catbaensis*.

2-methoxy-4-vinylphenol has been identified in red cabbage (*Brassica oleracea*) (in combination with other substances) and is associated with antibacterial properties and serving as insect repellent (Rubab et al. 2020). Eugenol (2-methoxy-4-(prop-2-en-1-yl)phenol) is predominantly found in the essential oils of several aromatic plants like clove (*Syzygium aromaticum*), nutmeg tree (*Myristica fragrans*), cinnamon (*Cinnamomum verum*) and basil (*Ocimum basilicum*) (Marchese et al. 2017). In plants, it serves multiple ecological roles, acting as a chemical defensive compound against pathogens and insects (Tian et al. 2015; Vargas-Méndez et al. 2019; Zhou et al. 2023; Hirose et al. 2024). Bassolé et al. (2010) observed that the efficacy of eugenol against pathogens is significantly enhanced when combined with other substances, particularly other phenolic compounds, due to synergistic effects that can reduce the concentrations required to inhibit microbial growth. Similar synergetic effects can be hypothesized for the chemical repertoire of *N. catbaensis*. Particularly noteworthy are the relatively low amounts of these two minor substances in the repellent secretion (2-methoxy-4-vinylphenol and eugenol). The quantity of both substances may only increase if required, i.e. in the presence of specific pathogens or other environmental triggers. Alternatively, the two components exclusively enhance synergetic effects. Thus, 2-methoxy-4-vinylphenol and eugenol could have a rather antimicrobial/antibacterial effect, and 4-vinylphenol might probably serve as a defensive component towards predators. However, it is more likely that the entire mixture of components fulfills multiple functions and that specific roles should not necessarily be attributed to individual components. Since the components in the secretion of *N. catbaensis* are unknown as defensive substances (besides functioning as insect repellent), it is unclear to which degree they are effective against predators or parasites. The harmful effects of 4-vinylphenol described by Carlson et al. (2002) are likely to be negligible, given the small amounts of the repellent secretion and the brief contact between potential predators and the insect. On humans, the secretion does not have an irritating effect on mucous membranes like other phasmatodean repellent secretions and might even be perceived as pleasant (pers. obs.). Nonetheless, the burnt and smoky odor alone might act as a deterrent, triggering a flight response in numerous animals.

Since all three substances occur in a variety of plants and phasmatodeans are exclusively herbivorous, it is reasonable to assume that *Neohirasea catbaensis* sequesters the components readily available from its food plant. It is obvious that no ubiquitous way of repellent secretion production exists given the high disparity of chemical substances. Happ et al.(1966) showed that *Anisomorpha buprestoides* produces its repellent chemicals de novo. In contrast, *Megacrania tsudai* sequesters the major component of the repellent secretion, actinidine, from its foodplant (Ho and Chow 1993). However, *Megacrania* is specialized in exclusively feeding on screw pines, whereas *Anisomorpha* is polyphagous and relies on a wide range of different plants (Brock and Büscher 2022). Merely for one species, *Oreophoetes peruana*, the synthesis routes for their repellent chemical (quinoline) are known: *Oreophoetes* is capable of converting L-tryptophan to quinoline via kynurenine and quinaldic acid as intermediates (Attygalle et al. 2021). L-tryptophan is an essential amino acid and involved in plant development, e.g., auxin synthesis (Radwanski and Last 1995), thus being indispensable for vascular plants. *Neohirasea* is not specialized in a specific food source and feeds on a variety of unrelated plants, e.g., *Rubus* spp. (bramble, Rosaceae), *Hedera* spp. (ivy, Araliaceae), *Quercus* spp. (oak, Fagaceae), *Hypericum* spp. (St. John’s Wort, Hypericaceae), *Eucalyptus* spp. (Myrtaceae) and ferns (Polypodiopsida) (Brock and Büscher 2022). Feeding different types of plants to our specimens did not result in any changes to the composition of the defensive secretion. It is therefore likely that the insects synthesize their repellent substances de novo from basic plant precursors. The observed compounds share common structural features. They likely all originate from ferulic acid and coumaric acid, which are key ubiquitous phenolic components linked to lignin and hemicellulose (Boerjan et al. 2003). In the case of 4-vinylphenol and 2-methoxy-4-vinylphenol, a simple decarboxylation step leads to the formation of these two compounds. Eugenol biosynthesis in plants is slightly more complex, but it also requires ferulic acid (Hanko et al. 2023). Due to the high concentration of 4-vinylphenol, it can be assumed that p-coumaric acid is either highly available in the diet or that the biosynthesis process is most efficient with this substrate and less efficient with ferulic acid.

The present study further contributes to our understanding of the unexpected and extraordinary chemical diversity of repellent substances in stick and leaf insects, underlining how incomplete our current knowledge still is. With only few species studied in detail, it remains unclear how defensive chemicals are distributed across the Phasmatodea tree of life and to what extent they reflect the group’s phylogeny, ecology, or life-history traits. A broader, phylogenetic approach, particularly within the species-rich Necrosciinae, will be necessary to test whether certain chemical compounds or gland types show evolutionary clustering or whether defensive chemistry has evolved in a species-specific manner. Another important unresolved aspect concerns the biosynthetic origin of the defensive compounds. The biosynthetic pathways underlying the production of repellent secretions in most phasmatodeans are still unknown, yet they are crucial for a comprehensive understanding of the evolution of such a wide variety of defensive components.

Future work should combine comparative chemical analyses with morphological investigations of the repellent glands to test for associations between glandular anatomy and the chemical components. In this context, the difference in relative gland size between male and female *Neohirasea catbaensis* is particularly intriguing and offers considerable potential for future research into sex-specific trade-offs.

## Data Availability

The morphological and chemical datasets generated in this study are available upon request from the corresponding author.
